# Potential Antitumor Activity and Apoptosis Induction of* Glossostemon bruguieri* Root Extract against Hepatocellular Carcinoma Cells

**DOI:** 10.1155/2017/7218562

**Published:** 2017-03-22

**Authors:** Mona S. Alwhibi, Mahmoud I. M. Khalil, Mohamed M. Ibrahim, Gehan A. El-Gaaly, Ahmed S. Sultan

**Affiliations:** ^1^Botany and Microbiology Department, Science College, King Saud University, P.O. Box 2455, Riyadh 11451, Saudi Arabia; ^2^Molecular Biology Unit, Zoology Department, Faculty of Science, Alexandria University, P.O. Box 21511, Alexandria, Egypt; ^3^Botany and Microbiology Department, Faculty of Science, Alexandria University, P.O. Box 21511, Alexandria, Egypt; ^4^Biochemistry Department, Faculty of Science, Alexandria University, P.O. Box 21511, Alexandria, Egypt; ^5^Oncology Department, Lombardi Comprehensive Cancer Center, Georgetown University Medical Center, Washington, DC 20057, USA

## Abstract

*Glossostemon bruguieri* (moghat) is used as a nutritive and demulcent drink. This study was performed to investigate the antiproliferative effects of moghat root extract (MRE) and its apoptotic mechanism in hepatocellular carcinoma (HCC) cells, HepG2 and Hep3B. MTT assay, morphological changes, apoptosis enzyme linked immunosorbent assay, caspase and apoptotic activation, flow cytometry, and immunoblot analysis were employed. The IC_50_ of MRE for HepG2 (910 ± 6 *μ*g/ml) and for Hep3B (1510 ± 5 *μ*g/ml) induced significant growth-inhibitory effects against HCC cells, with no cytotoxic effect on normal hepatocytes. MRE treatment induced apoptotic effects to HepG2 cells in a caspase-dependent manner and via upregulating p53/p21 and PCNA. The upregulation of p21 was controlled by p53 expression in HepG2 but not in Hep3B despite upregulation of Bax protein in both cell lines. Interestingly, p21 may be a remarkable switch to G1 arrest in HepG2 cells, but not in Hep3B cells. In addition, Fas- and mitochondria-mediated pathways were found to be involved in MRE-induced apoptosis in Hep3B cells. The GC-MS analysis of MRE revealed two major constituents of pharmaceutical importance: the flavonoid apigenin (17.04%) and the terpenoid squalene (11.32%). The data presented in this paper introduces* G. bruguieri* as a promising nontoxic herb with therapeutic potential for HCC. To the authors' knowledge, the present study provides the first report on the anticancer activity of MRE on HCC cells.

## 1. Introduction

Plants possess a number of biological and pharmacological activities including anticancer, anti-inflammatory, diuretic, oxytocic, laxative, antispasmodic, antihypertensive, antidiabetic, and antimicrobial functions. Numerous biological active products have been extracted from plants and have been used extensively as drugs, additives, flavours, insecticides, colorants, and fragrances [1]. In addition, the use of plants and plant-derived compounds for medicinal purposes is attracting interest as complementary and alternative therapies in many developed countries [2]. The plant with the tapered dark-colored roots,* Glossostemon bruguieri *(known as moghat in Egypt), is a shrub, briefly mentioned in Avicenna's* The Cannon of Medicine*, and one of the members of the cacao family Sterculiaceae [3].* G. bruguieri* grows wild in Iraq and Iran from where it was imported and introduced to Egypt in 1932 [4]. The hot syrup prepared from powdered moghat peeled roots is used for the treatment of spasms and as a mucoprotective agent [5]. Due to its high content of mucilage (35%), the syrup is customarily used by nursing mothers to induce lactation [6]. Other constituents isolated from moghat include proteins [7], estrone [8], amino acids, scopoletin [9], and the flavonoid takakin 8-*O*-glucoside [10]. Moreover, 80% of the total fatty acids extracted from moghat roots are oleic and linolenic acids. In addition, the roots possess major minerals (iron, calcium, and magnesium) and minor minerals (copper, zinc, and manganese) [11].

It is understandable that very few studies have investigated the biological effects of moghat root extract (MRE) since the plant is not well known in Western countries. In addition, as far as we know, reported studies did not include any investigation of the effect of MRE on different cancers. It was reported that MRE can lower the blood glucose level in diabetic rats by 54.5% in 15 days [9]. In addition, MRE decreased osteoclastic resorption and increased osteoblastic formation markers in rats with juvenile osteopenia [12]. Furthermore, emodin, a natural product from* G. bruguieri*, inhibited TNF-induced NF-kB activation, IkBa degradation, and expression of cell surface adhesion proteins in human vascular endothelial cells [13].

Hepatocellular carcinoma (HCC) has increased threefold during the last fifteen years in Egypt and topped the mortality statistics for cancers (23.2%) [14]. More than 500,000 individuals suffer from this disease annually and the front-line treatment for HCC is liver transplantation or resection [15]. Despite surgical or locoregional therapies, prognosis remains poor because of high tumor recurrence or tumor progression. Taking this into account and from the rationale that the uncommon ethnopharmacological uses of* G. bruguieri* deserve further study to investigate its biological activities, we aimed to evaluate the effect of MRE on HCC cells and HepG2 and Hep3B cell lines. In addition, we investigated the possible pathways by which MRE induces apoptosis in HCC cells.

## 2. Material and Methods

### 2.1. Materials

All the general purpose chemicals were purchased from Sigma-Aldrich, Thermo Fisher Scientific, and BDH AnalaR unless otherwise stated. General cell culture reagents were purchased from Lonza (Verviers, Belgium). FBS was purchased from HyClone (Thermo Fisher Scientific). HepG2 cell line was purchased from the American Type Culture Collection (Rockville, MD, USA). Moghat roots* (Glossostemon bruguieri)* were purchased from Egyptian local herbal market and authenticated by Professor M. Ibrahim, Botany and Microbiology Department, Faculty of Science, Alexandria University, Egypt.

### 2.2. Methods

#### 2.2.1. Preparation of Moghat Roots Extracts

*Moghat Roots Extract (MRE) Preparation.* Moghat roots were screened manually to remove bad ones. The dry roots were ground three times using an electric grinder. The powder was extracted in boiled sterilized distilled water, filtered, and concentrated with minor modification [16]. The extract was reconstituted in dimethyl sulfoxide (DMSO) to a working stock concentration of 50 mg/ml for further in vitro experiments.

*Moghat Roots Organic Extract Preparation for GC-MS Analysis. *20 gm of the power-driven screened dry moghat roots was successively extracted with n-hexane, chloroform, ethyl acetate, and alcohol (95%) separately using Soxhlet apparatus. The extracts were collected and concentrated in vacuum in a rotary evaporator at 50°C, and 2 *μ*l of the extract, containing both polar and nonpolar compounds, was used in GC-MS analysis.

#### 2.2.2. Cell Culture

HepG2 and Hep3B cells were maintained in DMEM supplemented with FBS (10%, v/v), penicillin (100 U/ml), and streptomycin (100 *μ*g/ml). Cells were maintained in 5% CO_2_ at 37°C. The confluent cells were passaged with trypsin-EDTA. Untreated medium containing vehicle DMSO was used as a negative control.

Normal liver specimens were collected with the informed consent of the patients, according to Georgetown University Institutional Review Board (Washington, DC) protocols. Normal primary liver human hepatocytes were established as previously reported [17] with some modifications (Sultan and Albanese; data under publication) to indefinitely extend the life span of primary human keratinocytes, using a Rho-associated kinase (ROCK) inhibitor, Y-27632 [18], with no feeder cells. Epithelial cells were cocultivated in F medium [3 : 1 (v/v) F-12 Nutrient Mixture (Ham)-Dulbecco's modified Eagle's medium (Invitrogen), 5% fetal bovine serum, 0.4 *μ*g/mL hydrocortisone (Sigma-Aldrich), 5 *μ*g/mL insulin (Sigma-Aldrich), 8.4 ng/mL cholera toxin (Sigma-Aldrich), 10 ng/mL epidermal growth factor (Invitrogen), and 24 *μ*g/mL adenine (Sigma-Aldrich)] with addition of 5 to 10 *μ*mol/L Y-27632 (Enzo Life Sciences).

#### 2.2.3. MTT Assay

HepG2 and Hep3B cells were cultured till midlog phase in a 96-well plate at a density of 2 × 10^4^ cells/well for 24 h prior to treatment with different concentrations (0 to 2000 *μ*g/ml) of MRE (control cells with vehicle only). The cells were washed twice with PBS after 48 h of incubation. Then MTT reagent (20 *μ*L of 5 mg/mL) (Promega, Madison, WI, USA) was added to each well. After 4 h incubation at 37°C, the medium was discarded and cells were incubated with 100 *μ*l of DMSO. The plate was shaken on a microvibrator for 5 min and the absorbance was measured at 570 nm. In order to achieve significant quantitative analysis, experiments were repeated at least 3 times in triplicate and compared with untreated control experiments.

#### 2.2.4. Cell Morphological Analysis

HepG2 cells were seeded into 12-well culture plates at a density of 2 × 10^5^ cells. After 24 h, cells were treated with fresh medium containing either 1/10 or 1/2 the calculated IC_50_ for MRE and incubated in 5% CO_2_ at 37°C. The mock sample received equal volume of medium with DMSO. After 48 h, treated and untreated cells were examined and cell images were taken using an inverted phase contrast microscope (Optika, Italy) at 200x magnification.

#### 2.2.5. Enzyme Linked Immunosorbent Apoptosis Assay

2 × 10^4^ Cells were seeded in a 96-well plate for 24 h. Media were changed to media containing MRE dose (1/10 or 1/2 the IC_50_). Cells were incubated for extra 24 h. To measure histone release from fragmented DNA in apoptosing cells Cell Death Detection ELISA PLUS Kit (Roche-Applied Science, Indianapolis, USA) was used. Cells were lysed with 200 *μ*L lysis buffer for 30 min at RT. The lysates were subjected to 10 min spin and 200 *μ*l of supernatant was collected, of which 20 *μ*l was incubated with antihistone biotin and anti-DNA peroxidase for 2 h at RT. After three washes with incubation buffer, 100 *μ*l of substrate solution 2,2′azino-di (3-ethylbenzthiazolin-sulphuric acid) was added to each well and incubated for 15–20 min at RT. The absorbance was measured using an ELISA reader (Jenway Spectrophotometer, UK) at 405 nm. Each assay was done in triplicate and the standard deviation was calculated.

#### 2.2.6. Caspase-3 Activity

Caspase-3 activity was assayed according to manufacturer's protocol (Assay designs, USA). 5 × 10^6^ cells were lysed in 100 *μ*l lysis buffer containing 10 mM HEPES (4-(2-hydroxyethyl)-1-piperazineethanesulfonic acid), pH 7.4, 2 mM EDTA, 0.1% CHAPS 3-[(3-cholamidopropyl)dimethylammonio]-1-propanesulfonic acid, 5 mM, 350 *μ*g/ml PMSF (phenylmethanesulfonylfluoride), and 5 mM DTT (dithiothreitol). Cells were homogenized by three cycles of freezing and thawing and then centrifuged to remove cellular debris. Each sample was incubated in buffer containing 10 mM HEPES, pH 7.4, 2 mM EDTA, 0.1% CHAPS, and 5 mM EDTA supplemented with its substrate (Ac-Asp-Glu-Val-Asp-AFC) Ac-DEVD-AFC for 1 h at room temperature and then the reaction was stopped with 1 N HCl. The absorbance at 405 was measured using a spectrophotometer (Jenway Spectrophotometer, UK). Each assay was done in triplicate.

#### 2.2.7. Western Blot Analysis

HepG2 and Hep3B cells were seeded into 6-well plates and were treated with fresh medium containing either 1/10 or 1/2 the calculated IC50 for MRE and incubated in 5% CO_2_ at 37°C. The mock sample received equal volume of medium with DMSO. After 48 h, cells were lysed in ice-cold RIPA lysis buffer supplemented with 1x protease inhibitors cocktail (Complete Mini®, Roche, Germany) and phosphatase inhibitors (Sigma) for 45 min on ice, followed by centrifugation at 10,000 g for 15 min at 4°C. The supernatant was harvested, and the protein concentrations were determined using the BCA assay (Pierce). Samples were mixed in a ratio of 1 : 2 in loading buffer and denatured by heating at 98°C for 5 min. 50 *μ*g/*μ*L of total proteins was separated on 10% Tris-SDS-PAGE gels (Bio-Rad Laboratories, USA) at 100 V for 1 h. The separated proteins were transferred to PVDF membrane (Bio-Rad Laboratories, USA) at 380 mA for 1 h. The membranes were blocked for 1 hour at room temperature with 5% skimmed milk in TBS (20 mM Tris-HCl, pH 7.6, and 137 mM NaCl) and probed overnight with the primary antibodies (1 : 1000) at 4°C. The following antibodies were used: p53, PCNA, p21, Fas, Bax, and PARP (Santa Cruz Biotechnology, CA, USA) and *β*-actin (Sigma). Blots were washed and incubated with horseradish peroxidase-conjugated secondary antibodies (Santa Cruz Biotechnology, CA, USA) for 1 h at room temperature. The immunoreactive bands of proteins were detected using ECL kit according to the manufacturer's instructions (Amersham, UK). Protein bands were quantified using Quantity One software (Bio-Rad Laboratories, USA). Protein levels were normalized against untreated control and *β*-actin.

#### 2.2.8. Gas Chromatography-Mass Spectrometry (GC-MS) Analysis

The GC-MS analysis of the alcoholic extract of* G. bruguieri* roots was carried out using a Perkin-Elmer GC Clarus 500 system (AutoSystem XL) comprising a Gas Chromatograph interfaced to a Turbo-Mass Gold-Perkin-Elmer mass-detector (GC-MS) equipped with Elite-1MS (100% dimethyl polysiloxane) fused capillary column (30 m × 0.25 mm ID × 1 *μ*m). For GC-MS detection, an electron ionization system was operated in electron impact mode with ionization energy of 70 eV. 99.99% Helium gas was used as a carrier gas at a constant flow rate of 1 ml/min, and the injection volume was employed at a split ratio of 10 : 1. The injector temperature was maintained at 250°C, the ion-source temperature was 200°C, and the oven temperature was programmed from 110°C (isothermal for 2 min), with an increase of 5°C/min to 280°C, ending with a 9 min isothermal at 280°C. Mass spectra were taken at 70 eV and the mass spectral scan range was set at 45–450 (*m*/*z*). The relative percentage of each component was calculated by equating its average peak area to the total areas. Turbo-Mass ver-5.2 software was adopted to handle mass spectra and chromatograms.

#### 2.2.9. Identification of Phytocompounds

The database of Wiley 275.1 MS library was used for interpretation on mass-spectrum of GC-MS. The spectrum of the unknown components was compared with the spectrum of known components stored in the Wiley 275.1 MS library. The name, molecular weight, and formula of the components of MRE were determined.

### 2.3. Statistical Analysis

The results were presented as the mean ± SD of at least three independent experiments. A one-way analysis of variance (ANOVA) was performed using the prism statistical package (GraphPad Software, USA).* P *value was calculated versus control cells: ^#^*P* < 0.01 and ^**∗**^*P* < 0.001 were considered statistically significant.

## 3. Results

### 3.1. MRE Inhibited Growth and Proliferation of HepG2 and Hep3B Cells but Not Normal Human Hepatocytes

**Figure 1:**
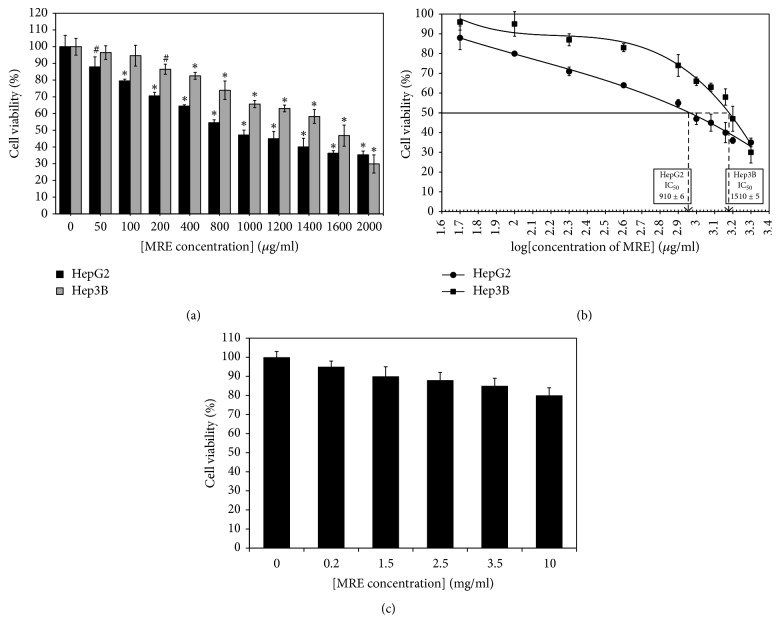
*Dose-dependent growth inhibition of HepG2 and Hep3B cells by MRE*. HepG2 and Hep3B cells were treated with MRE (0~2000 *μ*g/ml) for 48 hrs. Controls were defined as cells treated with 0.1% DMSO without MRE. (a) Effects of MRE on the proliferation of HepG2 and Hep3B cells were expressed as percentage of cell viability. (b) Effects of 48 hrs exposure to varying concentrations of MRE on cell proliferation are expressed as concentrations of MRE at which the viability of cells can be reduced to 50% (IC_50_). (c) In vitro cytotoxicity of the MRE against normal human hepatocytes; a nonviral normal human liver epithelial cell line were treated by different concentration of the MRE. Data from at least three independent experiments performed in at least triplicate are presented as means ± SD;* P *value was calculated versus control cells: ^#^*P* < 0.01 and ^*∗*^*P* < 0.001.

To explore the growth-inhibitory potency of MRE on hepatocellular cells, cell proliferation was determined by MTT assay. The cytotoxic effects of MRE on HepG2 and Hep3B cells were determined by treating cells with varying concentrations of MRE (0–2000 *μ*g/ml) for 48 hrs. In addition, normal liver hepatocytes were treated with different concentrations of MRE (0–10 mg/ml) for 48 hrs. The data indicate that treatment of cells with increasing concentrations of MRE resulted in significant inhibition of cell viability in a dose-dependent manner when compared to untreated controls (Figure 1(a)). MRE was found to inhibit the proliferation of HepG2 and Hep3B cells in a dose-dependent manner. After 48 hours of exposure to MRE, 65% of the HepG2 cells and 70% of the Hep3B cells were observed to be dead at the highest concentration (2 mg/*μ*L). Growth was inhibited even at lower concentrations of MRE. The concentration at which 50% inhibition of cell viability (IC_50_) was calculated using semilogarithmic plotting of the percentage of cell viability versus concentration used for MRE. The IC_50_ of MRE for HepG2 (910 ± 6 *μ*g/ml) and for Hep3B (1510 ± 5 *μ*g/ml) induced a significant decrease in survival of both cell lines when compared to untreated control after 48 hrs treatment (Figure 1(b)). Normal hepatocytes showed no significant antiproliferative effect for MRE treatment (Figure 1(c)). For subsequent experiments two different concentrations for MRE will be employed as follows: 91 and 455 *μ*g/ml for HepG2 cells and 151 and 755 *μ*g/ml for Hep3B cells, which represent 1/10 and 1/2 MRE IC_50_, respectively.

### 3.2. MRE Induced Morphological Changes in HepG2 Cells

**Figure 2:**
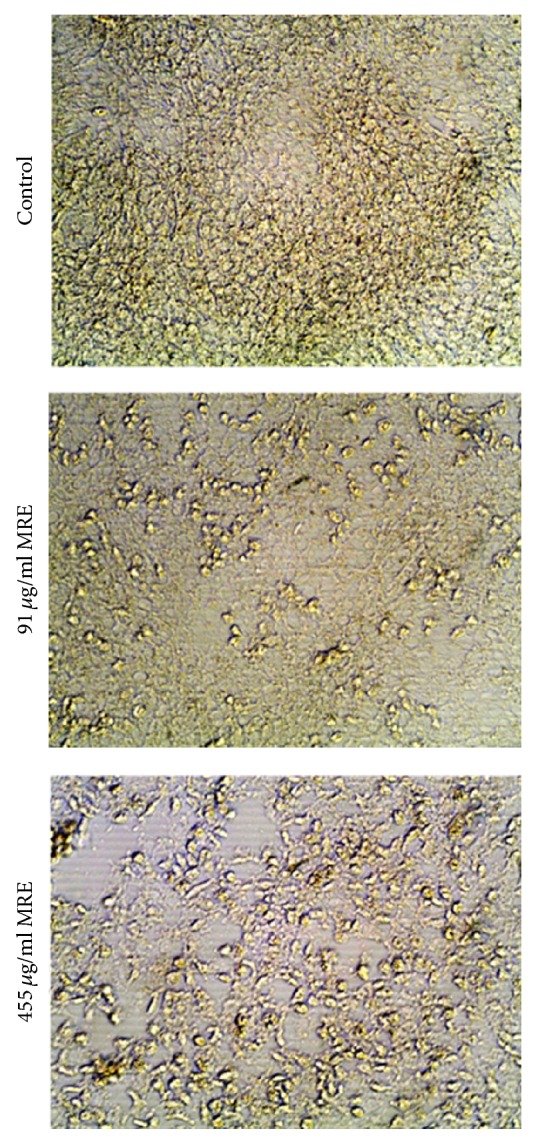
*MRE induced morphological alterations in HepG2 cells*. HepG2 cells were either untreated or treated with MRE at different concentrations (91 and 455 *μ*g/ml) for 48 hrs. Cell images were taken using an inverted microscope at 200x magnification. Experiment has been performed in triplicate.

To examine the effect of MRE on the morphology of HepG2 cells, cells were cultured and treated for 48 hrs with 91 or 455 *μ*g/ml MRE. As shown in Figure 2, microscopic examination of the treated cells showed that the treatment with the higher concentration of MRE resulted in dramatic morphological changes in HepG2 cells. We observed changes in the morphology of cells from elongated and spindle shape to rounded and epithelial-like shape. The monolayer cells became rounded up, lost contact with neighboring cells, and were largely detaching from culture plate. 48 hrs posttreatment, cells showed altered normal morphology, shrinkage, tendency to float in the medium, and reduction in size in comparison to the untreated controls that were well spread with a flattened morphology. Taken together MRE induced morphological changes in the hepatocellular carcinoma HepG2 cell line.

### 3.3. Apoptosis Is Induced in MRE-Treated HepG2 Cells

**Figure 3:**
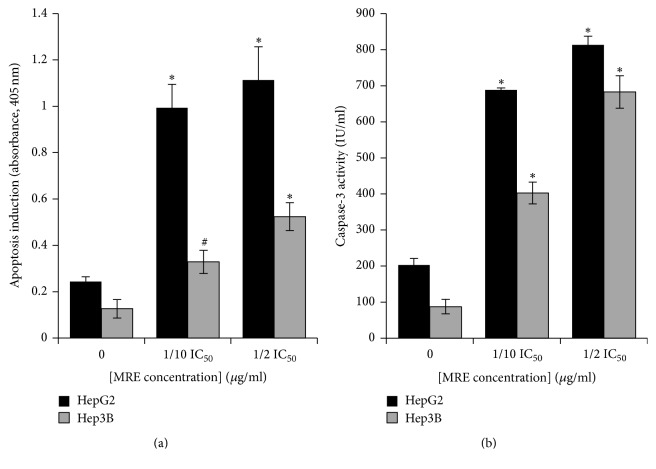
*MRE induced apoptosis in HepG2 and Hep3B cells in a caspase-dependent manner*. Cells were either untreated or treated with MRE at different concentrations (1/10 and 1/2 IC_50_) for 48 hrs. Lysed cells were subjected to (a) enzyme linked immunosorbent apoptosis assay to measure the histone release as an indication for apoptosis and (b) caspase-3 activity assay. Each assay was done in triplicate and standard deviation was calculated. Data are presented as mean ± SD.* P *value was calculated versus control cells: ^#^*P* < 0.01 and ^*∗*^*P* < 0.001.

Our data indicated that MRE significantly decreased cell viability and significantly altered the morphology of HepG2 cells after MRE treatment compared to control untreated cells (Figures 1 and 2). To investigate if the decrease in the viability of cells was due to induced-apoptosis, enzyme linked immunosorbent apoptosis assay was performed, which detects histone release from apoptotic cells. As shown in Figure 3(a), 48 hrs treatment of HepG2 with either 91 or 455 *μ*g/ml MRE confirmed that the tested MRE-induced cell death was due to apoptosis as indicated by histone release when compared to untreated control cells.

### 3.4. MRE Induced Caspase-3 Activation in HepG2 Cells

Our data indicated that caspase-3 activity was significantly increased in MRE-treated HepG2 cells when compared to control cells. As shown in Figure 3(b), MRE at the apoptosis-inducing concentrations (91 and 455 *μ*g/ml) significantly induced caspase-3-like activity in HepG2 cells. These results demonstrate that the active tested MRE induced apoptosis of HepG2 cells in a caspase-dependent manner.

### 3.5. MRE Induced G1 Phase Arrest in HepG2 Cells

**Figure 4:**
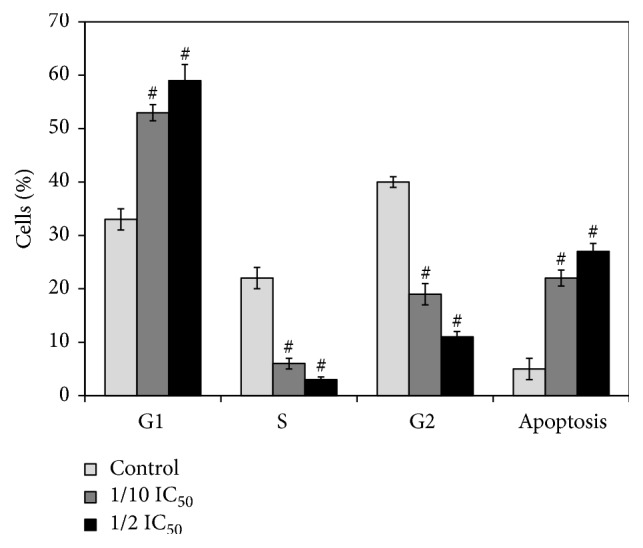
*Cell cycle analysis of HepG2 cells treated with MRE*. The percentage of cell cycle phases was determined in HepG2 cells treated with or without the MRE for 48 hrs compared to the mock-treated control. 1/10 MRE IC_50_ = 91 *μ*g/ml and 1/2 MRE IC_50_ = 455 *μ*g/ml. Each assay was done in triplicate and standard deviation was calculated. Data are presented as mean ± SD.* P *value was calculated versus control cells: ^#^*P* < 0.01.

To determine whether MRE's growth-inhibitory effect was caused by specifically perturbing cell cycle-related events, cell cycle analysis by flow cytometry analysis was carried out.  Figure 4 shows the relative percentages of HepG2 cells in each phase of the cell cycle, following 48 hrs treatment with different MRE concentrations. MRE treatment induced the accumulation of G1 phase of cell cycle in a concentration-dependent manner in HepG2 cells with increase of subG1 population after MRE treatment with 1/10 and 1/2 MRE IC_50_, respectively. This data suggested that the growth inhibition of HepG2 cells was the result of a G1 phase arrest and through apoptosis induction.

### 3.6. MRE Induced p21 and G1 Arrest in HepG2 in a p53-Dependent Manner

**Figure 5:**
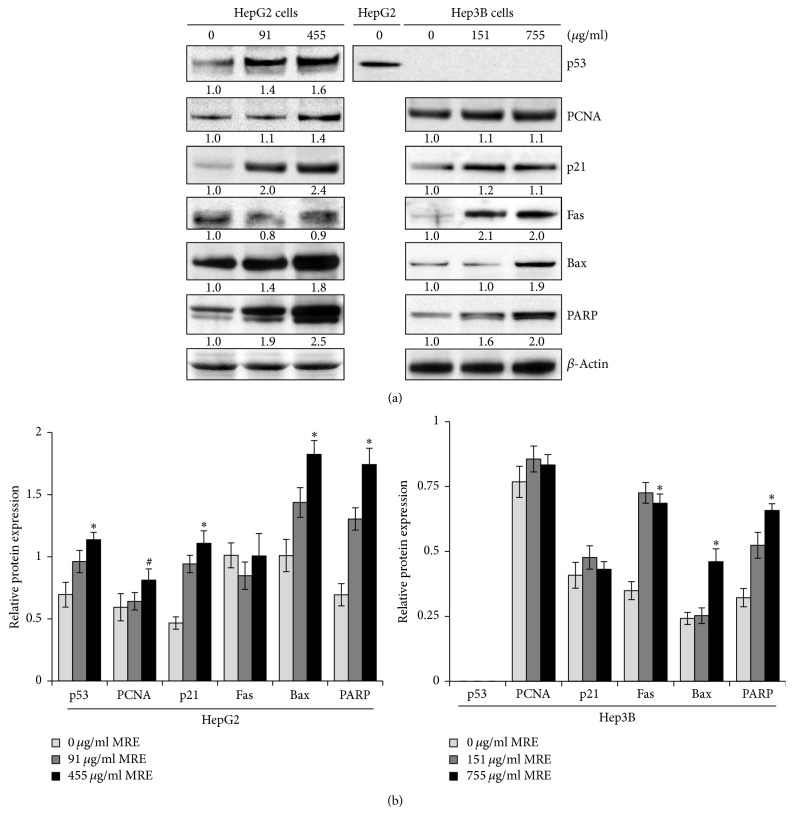
*The effect of MRE treatment on p53, PCNA, p21, Fas, Bax, and PARP expressions*. (a) Protein expression levels were assessed by Western blot analysis in HepG2 and Hep3B cells after exposing cells with the indicated concentrations of MRE for 48 h. 50 *μ*g of total cell lysate from each sample was subjected to 10% SDS-PAGE. Proteins were transferred to PVDF and probed with the indicated antibodies. Anti-*β*-actin was used as a loading control. Protein expressions were quantified, normalized to *β*-actin and controls. Numbers represent fold of change. (b) Relative protein expression levels in HepG2 and Hep3B cells were quantified using Quantity One software. The experiment was done in triplicate. Data are presented as mean ± SD.* P *value was calculated versus control cells: ^#^*P* < 0.01 and ^*∗*^*P* < 0.001.

Since it was reported that the tumor-suppressor p53 regulates a DNA-damage-triggered G1 checkpoint by upregulation of CDK inhibitor p21, we examined the expression patterns of p53 and p21 after MRE treatment. As shown in Figure 5, HepG2 (wild-type p53) cells treated with MRE showed an increase in the protein expression of p53 and p21 in a concentration-dependent manner. In contrast, Hep3B cells treated with MRE showed no p53 protein expression with no changes in the protein levels of p21 after 48 hrs. In addition, the protein expression levels of PCNA were examined by Western blot analysis in MRE-treated HepG2 cells. PCNA protein expression was upregulated only in HepG2 cells with the treatment with the higher concentration of MRE (455 *μ*g/ml), but there was no changes in the protein expression levels of PCNA in case of Hep3B cells. The inhibitory effect of MRE treatment on Hep3B cells may involve another mechanism rather than p53 and p21.

### 3.7. MRE Triggered Apoptosis in Hep3B in a p53-Independent Manner

We investigated whether the expressions of Fas, Bax, and PARP were modulated by MRE treatment. The treatment of Hep3B cells (expressing no p53) with MRE resulted in a concentration-dependent increase in the expression of Fas, but not in HepG2 cells. In addition, MRE treatment significantly increased Bax expression in a concentration-dependent manner in both HepG2 and Hep3B cells. PARP cleavage was also measured in MRE-treated HCC cells. A significant increase of cleavage forms of PARP was observed in both cell lines in a concentration-dependent manner (Figure 5).

### 3.8. GC-MS Analysis of* Glossostemon bruguieri* Roots Organic Extract

**Figure 6:**
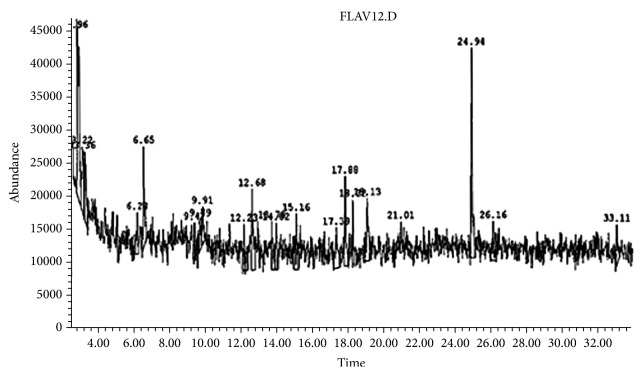
Chromatogram of the GC-MS analysis of the organic extract* of Glossostemon bruguieri* roots.

The GC-MS analysis of the organic extract of* G. bruguieri* roots is resolved into 21 peaks. As shown in Figure 6, in terms of percentage peak area, ten major peaks were determined in the GC-MS chromatogram. The compounds, corresponding to the 21 peaks, were compared with Wiley 275.1 MS library and the compounds' name, retention time, molecular formula, molecular weight, and concentration (peak area%) are presented in Table 1. These compounds were mostly flavonoids (38.9%), terpenoids (26.15%), and fatty acid derivatives (16.2%). Two major constituents were identified in the extract: the flavonoid apigenin (17.04%) and the terpenoid squalene (11.32%). The remaining components were considered as minor constituents and ranged between 0.8% for the phenolic compound chlorogenic acid and 6.49% for the flavonoid kaempferol.

## 4. Discussion

Currently there is a mounting interest in the use of phytocompounds to develop safe and more effective therapeutic agents for cancer treatment [19, 20]. Phytochemicals are a promising group of potential cancer chemopreventive agents because of their low toxicity and their important role in treatment and prevention of human diseases [21, 22]. Phytochemical antioxidative and anti-inflammatory activities have demonstrated potent anticancer activity in multistage carcinogenesis [23].

*G. Bruguieri* (moghat), family Sterculiaceae, grows wild in Iraq and Iran from where it was imported and introduced to Egypt in 1932 [24]. It is considered as a remarkable food supplement and possesses significant hypoglycemic activity [9]. On the basis of dry matter, moghat roots contain 35% mucilage, 24% starch, 5.5% protein, 5% fats, 5% pectin, 3% sugars, and amino acids (majorly aspartic and glutamic acids) [25]. They also contain major minerals (calcium, iron, and magnesium) and minor minerals (zinc, copper, and manganese), as well as high amounts of oleic and linolenic acids [11]. In Egypt, sundried, powdered moghat roots are given in the form of hot syrup to nursing mothers as a nutritive and demulcent drink to stimulate lactation. The powdered roots are cooked with butter, sugars, nuts, and coconut in boiling water [6]. It was reported that the powder and alcoholic extract of* G. Bruguieri* roots when administrated orally to mice proved to be nontoxic up to 2500 mg/kg body weight [9]. Also, the acute and subchronic toxicity of the aqueous moghat extract were evaluated. The LD_50_ was higher than 10 g/kg in an acute toxicity study. In a subchronic toxicity study, doses of 200–1000 mg/ml had no hepato- and/or nephrotoxicity while the dose of 2000 mg/ml showed slight hepato- and nephrotoxicity [12]. To our knowledge, the antineoplastic effects of moghat root extracts (MRE) are not well documented, and this is the first study to demonstrate MRE apoptotic effects on HCC cells.

In the present study, we investigated the role of MRE on cell death for two HCC cell lines, HepG2 and Hep3B cells. MRE induced G1 cell cycle arrest and cell proliferation inhibition in HCC cells. Our data showed that the viability of HepG2 and Hep3B cells was significantly reduced in a concentration-dependent manner after MRE treatment. MRE treatment exhibited significant cytotoxicity reaching the maximum effect with treatment with the highest concentration of MRE (65% and 70% death in HepG2 and Hep3B cells, resp.). No cytotoxic effect on normal human hepatocytes was observed.

The results of the enzyme linked immunosorbent apoptosis assay on HCC cells after MRE treatment revealed significant induction of apoptosis. The activation of caspases, intracellular cysteine proteases, is one of the key events in apoptosis [26]. The typical morphological and biochemical characteristics observed in apoptosis are attributed in part to the cleavage of various substrates by caspases. Caspase-3 is a general mediator of physiological and stress-induced apoptosis [27]. Here, the activity of caspase-3 increased significantly in MRE-treated HCC cells. In agreement, the phytochemical diosgenin showed a chemopreventive effect against several human cancer cells via the activation of p53 and the modulation of caspase-3 activity [28]. In addition, the methanolic extract of black cumin* (Nigella sativa)* induced apoptosis in breast cancer cell line via p53 and caspases [29] and induced cytotoxicity against lung cancer cell line [30]. Furthermore, diosgenin and the phytochemical thymoquinone isolated from fenugreek* (Trigonella foenum-graecum)* and* Nigella sativa*, respectively, induced activation of caspase-3 through the stimulation of mitochondrial cytochrome C release in squamous cell carcinoma [31]. The same observations were reported in HL60 cells undergoing thymoquinone-induced apoptosis [32]. Taken together, these results demonstrate that the active tested MRE induced apoptosis in HCC cells in a caspase-dependent manner.

Being equipped with the tumor-suppressor and transcription factor activities explains the critical role of p53 in many cellular processes including DNA-damage response, genomic stability, cell cycle control, and apoptosis. p53 inhibits the growth of DNA-damaged cells and cancer cells by inducing apoptosis through activating the transcription of downstream apoptosis-related genes such as p21^cip1/waf1^ and Bax [33]. Many studies reported the treatment with phytochemicals, in different experimental model systems, such as the green tea polyphenol and epigallocatechin-3-gallate (EGCG) in HepG2 cells and LNCaP cells [34, 35], curcumin in human breast cancer cells and neuroblastoma [36, 37], resveratrol in neuroblastoma and thyroid carcinoma cell lines [37, 38], and diosgenin in human colon and lung cancer cell lines [39, 40]. These phytochemicals have been shown to suppress cell progression and induce apoptosis mainly by increasing the expression of p53 protein. Since p53 has been shown to be upregulated in different tumor cells after treatment with phytochemicals, we examined whether MRE could affect the expression of p53. In agreement with earlier reports, we found that MRE upregulated p53 protein level after treatment for 48 h, suggesting that p53 upregulation may be responsible for the inhibition of cell viability and apoptosis induction in treated HepG2 cells which in turn could be associated with the regulation of cell cycle-related p53 gene. In disagreement with our result and although curcumin is a powerful inhibitor of tumor cell proliferation, however, it induced growth arrest and apoptosis of B cell lymphoma and human melanoma cells by downregulating p53 (p53-independent mechanism) [41]. As reported previously, HepG2 cells exhibit a wild-type inducible p53 activity [42], which in turn may explain the involvement of the upregulation of p53 in the MRE-induced apoptosis in HepG2 cells.

Interestingly, despite the foregoing, treatment with MRE has an inhibitory effect on Hep3B cells, the inhibitory mechanism may not involve the P53/p21 axis, and another mechanism may be involved in the inhibitory process. Our data demonstrated that p53 and p21 might be crucial for MRE-induced apoptosis in HepG2 but not in Hep3B cells. The results from the comparative study between HepG2 and Hep3B cells suggested that MRE induced cell death through p53-dependent mechanism in HepG2 cells. This was not the case for Hep3B cells that showed no p53 expression, and in turn the expression level of p21 was not altered despite significant increases in Bax and PARP expression levels. This suggests that MRE may be able to induce cell death and apoptosis in Hep3B cells through a p53-independent mechanism.

PCNA is a crucial factor in maintaining the balance between survival and cell death [43]. Here in this study we aimed to compare the total protein levels of PCNA and the proliferative states of MRE-treated cells with the untreated control cells. The results here show that MRE-treated HepG2 exhibited upregulated expression of p53, p21, and PCNA when compared to the untreated controls. Interestingly, Hep3B cells exhibited no alteration in PCNA expression levels after MRE treatment. PCNA is involved in DNA repair and serves as a cofactor in S-phase for DNA polymerase delta [44]. PCNA has been reported to participate in the regulation of apoptosis, either by suppressing antiapoptotic proteins, including the Gadd45 family (Gadd45, Myd118, and CR6) in somatic cells, or by promoting proapoptotic proteins, such as ING1 [45–47]. The competitive binding of ING1 to PCNA through a site used by growth regulatory and DNA-damage proteins has been shown to induce apoptosis [46]. Although MCL1 (ML1 myeloid cell leukemia 1) is known to function as an antiapoptotic protein, it was demonstrated that the overexpression of MCL1, the only Bcl-2 family protein to interact with PCNA, decreased BrdUrd uptake, in HeLa cells, in a PCNA-dependent fashion. In this context, the upregulation of MCL1 arrested cell cycle progression via interaction with PCNA and prevented cells from replicating altered DNA. The mechanism may involve the use of a different region within the MLC1 molecule to facilitate apoptosis [48]. Moreover, the reduction in the PCNA content by siRNA significantly reduced the levels of PCNA–MutS*α*–MutL*α* protein complex that form on damaged DNA in human cells exposed to alkylating agents. PCNA siRNA treatment suppressed the increase in the caspase-3 activity, a hallmark for the induction of apoptosis [49]. Additionally, PCNA gene expression was upregulated in immortalized human endothelial cells undergoing p53-mediated apoptosis [50]. Other studies reported the involvement of the upregulation of PCNA with apoptosis in normal cells. When the expressions of apoptosis-related genes, after PCNA RNAi in mouse oocytes, were investigated, the mRNA levels of Bax, caspase-3, and TNF*α* decreased significantly accompanied by increased expression of Bcl-2. Also, all the autophagy markers that were tested (LC3I, LC3II, Atg5, and Becn1) did not show any changes after PCNA RNAi. These results indicated that the increase in oocytes in PCNA RNAi ovaries resulted from decreased oocyte apoptosis rather than autophagy [51].

P21 is p53 inducible and is a general cyclin-dependent protein kinase inhibitor [33]. In addition, it was reported that the human PCNA promoter can be transactivated by the wild-type p53 [52]. p21 is capable of competing with PCNA-binding proteins to disrupt DNA replication/repair machinery and to inhibit cell proliferation [53]. The p21-PCNA association does not affect the overall structure of PCNA or the PCNA-DNA association [54]. The binding of p21 to PCNA would interfere with the function of PCNA to synthesize DNA effectively, thereby impeding cell cycle progression [55]. Other studies have suggested that p21 is required to assist disassembly of PCNA from repair sites [56]. p21 inhibits DNA synthesis not only by binding to PCNA [55, 57] but also by binding to the CDKs [58]. It was shown that the base excision repair (BER) activity was induced and accompanied by the upregulation of p53 and PCNA in human colon cancer cell lines treated with the naturally occurring selenomethionine. The mechanism is p53-dependent and requires the binding of Gadd45a with PCNA and the endonuclease APE1/Ref-1 [59]. Gadd45a, the growth arrest and DNA-damage-inducible protein 45A, is one of the p53-activated downstream genes [60]. The activity of Gadd45a can be decided by its binding partners, and the binding of either PCNA/Cdc2 or p21 to Gadd45a can mediate cell cycle arrest or DNA repair, respectively [61]. Additionally, the human p57 protein, like p21, contains a PCNA-binding domain within its C terminus. The p57-PCNA complex can prevent DNA replication in vitro and S-phase entry in vivo. Thus, the control of cell cycle by p57 requires PCNA inhibitory activity, and disruption of this function may lead to uncontrolled cell growth [62].

To explore the possible apoptotic mechanism induced by MRE in Hep3B cells, we investigated the expression pattern of Fas, Bax, and PARP proteins in HepG2 and Hep3B cells after MRE treatment. We found that MRE-induced apoptosis was associated with mitochondria-mediated pathway in Hep3B including the upregulation of Bax, caspase-3 activation, and PARP. Additionally, MRE also activated Fas-mediated apoptosis pathway as evident by upregulation in Fas expression in Hep3B, not in HepG2 cells. MRE may have induced the release of cytochrome c and caspase-3 activation as reported previously [63]. Activated caspase-3 subsequently cleaves PARP, which serves as a hallmark of apoptosis [64]. These results suggest that MRE induced apoptosis of Hep3B cells via a mitochondria-mediated intrinsic apoptosis pathway. It was reported that the binding of Fas receptor to its ligand and the activation of caspases, including caspase-3, forms a death-inducing signal complex efficient in killing tumor cells and inducing apoptosis [65, 66]. The present study showed that the protein expression of Fas was upregulated and caspase-3 was activated with MRE treatment. These results suggest that a Fas-mediated extrinsic pathway might play a critical role in MRE-induced apoptosis in Hep3B cells. Taken together, these data suggest that MRE could induce apoptosis of Hep3B cells through mitochondria- and Fas-mediated caspase-dependent pathways. These mechanisms have been reported previously using different agents to induce apoptosis of HCC cells [67–69].

Venturi et al. reported the detection of two PCNA expression patterns in human liver tumors and cell lines [70]. The different pools of PCNA might act together and at the same time to carry out different functions within different signaling pathways, as reported previously [71]. Taken together, during cell cycle arrest, PCNA protein may form complexes with several proteins (p21, p57, Gadd45, MyD118, CR6, ING1, MCL1, MutS*α*, and MutL*α*). PCNA functions and interactions are modulated by posttranslational regulation which is thought to guide PCNA to the correct partner protein at the correct time to perform a specific function [70]. Studies suggest that PCNA is the supervisory molecule in life of the cell, forcing it to replicate DNA, arrest the cell in G1 or G2 phase, repair damaged DNA, or obligate the cell to apoptosis, based on the interactions with a plethora of proteins [72]. It was speculated that an increase in the expression of PCNA may create a disorder in the signals involved with DNA synthesis. This disorder may result in mitotic catastrophe and induction of apoptosis [73]. Mechanistically, it was reported that the histone lysine methyltransferase SETD8 promoted carcinogenesis by deregulating PCNA expression [43]. Furthermore, PCNA-MutS*α*-mediated binding of MutL*α* to replicative DNA with mismatched bases to induce apoptosis, a mechanism that may explain how p21 retained its function of cell cycle arrest while PCNA is occupied by the interaction with another protein partner and preventing cells from replicating altered DNA. How does PCNA fulfill its function as an executor of apoptosis? How the complexes that form between different binding partners and PCNA are recognized by the cell? And many more questions when answered will shed more light on PCNA regulation on the global cell life.

Based on the high antiproliferative activity of MRE against HCC cells, the organic extract of moghat roots was subjected to GC-MS analysis to analyze its constituents. 21 compounds have been identified from the organic extract of* G. bruguieri* roots by GC-MS analysis. The prevalent compounds were flavonoids, terpenoids, and fatty acid derivatives. Two major constituents were identified from the GC-MS results: apigenin and squalene. Very recently, a number of studies have focused on the activity of apigenin. These studies reported different activities for the flavonoid: antileukemic activities [74], increased chemosensitivity of cancer cells to drugs [75], anti-inflammatory activities [76], inhibition of nitric oxide production [77], and immune balance during inflammation [78]. In addition, apigenin was presented as a promising molecule for cancer prevention [79]. Furthermore, apigenin possesses antiatherogenic and vasoprotective properties [80, 81]. For squalene, it is known to be involved in the biosynthesis of phytosterol or cholesterol in plants or animals [82]. It was reported that squalene enhances the adaptive immune responses, as an adjuvant, by helping to generate antigen-specific T cells via antigen-carrying neutrophils and IL-18 [83]. In addition, it was reported that, after oral administration of 100 mg/kg* Flos Chrysanthemi* extract in rats, apigenin exhibited AUC_(0–12)_, *t*_1/2_*z*, and *C*_max_ equal to 36.25 ± 16 mg/lh, 18.44 ± 12 h, and 3.310 ± 0.73 mg/l, respectively [84]. Squalene, in a vaccine formulation, can assist in eliciting a stronger immune response while maintaining an adequate reactogenicity and safety profile [85]. Recently squalene-containing oil-in-water emulsion adjuvants have been approved for use in certain influenza vaccines [86]. In addition, squalene was also reported to have chemopreventive, antioxidant, pesticide, and sunscreen activities [87]. Also, it was reported that 60–85% of dietary squalene can be absorbed from an oral dose. Up to 90% of the postabsorptive dose is transported in serum until it is distributed ubiquitously in human tissue [88]. Rather than their therapeutic effects, plants are mostly used for food consumption. The phytochemical content of a particular plant, although it is genetically determined, also very much depends on the growing environment [89]. It must be noted that the phytochemical extraction protocol could affect the activity of extracted constituents according to previous observations. In the same context, some of the compounds of the hexane extract of fenugreek have been found to be more active in intrinsic apoptotic pathway rather than extrinsic pathway, which might be due to the presence/absence of methanolic soluble compounds [90].

## 5. Conclusions

Overall, our study demonstrates that MRE induced G1 phase arrest and apoptosis induction through p53/p21 axis and upregulation of PCNA signaling in a caspase-dependent manner in HepG2 cells, suggesting that p53/p21 might be a key axis in the G1 cell cycle arrest of HepG2 cells but not in Hep3B cells (with no p53 expression). In addition, Fas- and mitochondria-mediated pathways were found to be involved in MRE-induced apoptosis in Hep3B cells. Our findings suggest that moghat roots may have some active constituents that may have a potential antitumor effect, and further investigations on the phytoconstituents identified in the organic extract of* G. bruguieri* roots may add new knowledge to prove the medicinal importance of this plant in the future. In addition, the GC-MS analysis of moghat roots organic extract identified some constituents that were previously reported to possess an antitumor effect. We also aimed in this study to prepare the plant extract by the same method customarily used by nursing mothers in Egypt. How much of the moghat dietary constituents are absorbed and how they are metabolized and excreted need more investigation. Phytochemicals hold promise for cancer chemoprevention, but it must be noted that it is difficult to theorize and generalize data obtained from one study to another because the concentration of active constituents in any extract prepared from plants will differ according to a number of factors like geographical region, condition of soil, harvest season, degree of maturation of the plant, extraction protocol, and so forth [91]. To our knowledge, there is no single study on the liver cancer inhibitory properties of MRE constituents. The findings here along with future studies may introduce moghat roots as herbals with anticancer properties.

## Figures and Tables

**Table 1:** Phytochemical compounds identified in the GC-MS analysis of the organic extract of *Glossostemon bruguieri* roots.

PK	RT	Peak area (%)	Name of the compound	Molecular formula	MW	Nature of the compound
1	2.96	17.1	Apigenin	C_15_H_10_O_5_	270	Flavonoids
2	3.26	6.49	Kaempferol	C_15_H_10_O_6_	286	Flavonoids
3	3.36	5.91	Palmitic acid	C_16_H_32_O_2_	256	Fatty acids
4	6.28	2.15	Ethyl Palmitate	C_18_H_36_O_2_	284	Fatty acid esters
5	6.65	5.27	Salvigenin	C_18_H_16_O_6_	328	Flavonoids
6	9.46	0.81	Chlorogenic Acid	C_16_H_18_O_9_	354	Phenolic compounds
7	9.78	1.71	Heptanone	C_8_H_16_O	128	Ketone
8	9.91	2.34	Phytol	C_20_H_40_O	296	Precursor for vitamins E and K
9	12.2	3.83	Phthalic acid	C_28_H_46_O_4_	446	Dicarboxylic acid
10	12.7	5.66	Dibutyl phthalate	C_16_H_22_O_4_	278	Bisphenol
11	13.8	3.98	7-Methoxyflavone	C_28_H_46_O_4_	252	Flavonoids
12	14.1	2.84	Hydrocinnamic acid	C_9_H_10_O_2_	150	Phenylpropanoids
13	15.2	5.45	Pinene	C_10_H_16_	136	Monoterpene
14	17.4	4.65	Carvone	C_10_H_14_O	152	Terpenoids
15	17.9	4.73	Physcion	C_10_H_16_O_5_	284	Terpenoids
16	18.3	2.68	Methyl linolenate	C_19_H_32_O_2_	292	Fatty acids
17	19.2	6.12	Biflavonoid	(C_6_-C_3_-C_6_)_2_	538	Flavonoids
18	21.1	1.57	Chrysophanol	C_15_H_10_O_4_	254	Phenolic compound
19	24.9	11.3	Squalene	C_30_H_50_	410	Terpenoids
20	26.2	3.24	*α*-Linolenic acid	C_19_H_32_O_2_	292	Fatty acids
21	33.1	2.23	Icosatrienoate	C_21_H_36_O_2_	320	Fatty acids

PK: peak number; RT: retention time; MW: molecular weight.

## References

[1] Ebere Okwu D., Uchenna Nnamdi F. (2011). A novel antimicrobial phenanthrene alkaloid from *Bryopyllum pinnatum*. *Journal of Chemistry*.

[2] Sowemimo A. A., Fakoya F. A., Awopetu I., Omobuwajo O. R., Adesanya S. A. (2007). Toxicity and mutagenic activity of some selected Nigerian plants. *Journal of Ethnopharmacology*.

[3] Purseglove J. W. (1968). *Tropical Crops. Dicotyledons Volumes 1 and 2*.

[4] Shabatai Y., Osman I. (1939). *Cultivation of Moghat Plant in Egypt*.

[5] Sina I., Ali A. (1993). *Al Qanoon fil Tibb*.

[6] Meselhy M. R. (2003). Constituents from Moghat, the roots of Glossostemon bruguieri (Desf.). *Molecules*.

[7] Ibrahim N., El-Eraky W., El-Gengaihi S., Shalaby A. S. (1997). Chemical and biological evaluation of proteins and mucilages from roots and seeds of *Glossostemon bruguieri* Desf. (Moghat). *Plant Foods for Human Nutrition*.

[8] Amin E. S., Awad O., El Samad M. A., Iskander M. N. (1969). Isolation of estrone from moghat roots and from pollen grains of egyptian date palm. *Phytochemistry*.

[9] El-Sayed N. H., Awaad A. S., Mabry T. J. (2004). Phytochemical studies and effect on urine volume of Glossostemon bruguieri Desf. constituents. *Indian Journal of Experimental Biology*.

[10] Sharaf M., El-Ansari M. A., Saleh N. A. M. (1998). A new flavonoid from the roots of Glossostemon bruguieri. *Fitoterapia*.

[11] Gamel T. H., El-Razek A. M. A., Damir A. A. (2010). Dried peeled roots of glossostemon bruguieri (moghat) as a potential functional food. *Journal of Food Processing and Preservation*.

[12] Ghareeb D. A., El-Rashidy F. H., El-Mallawany S. (2014). Imbalanced diet deficient in calcium and vitamin D-induced juvenile osteopenia in rats; the potential therapeutic effect of Egyptian Moghat roots water extract (*Glossostemon bruguieri*). *Iranian Journal of Pharmaceutical Research*.

[13] Takada Y., Aggarwal B. B. (2003). Betulinic acid suppresses carcinogen-induced NF-*κ*B activation through inhibition of I*κ*B*α* kinase and p65 phosphorylation: abrogation of cyclooxygenase-2 and matrix metalloprotease-9. *Journal of Immunology*.

[14] Ferlay J., Soerjomataram I., Dikshit R. (2015). Cancer incidence and mortality worldwide: sources, methods and major patterns in GLOBOCAN 2012. *International Journal of Cancer*.

[15] Lee K. K., Kim D. G., Moon I. S., Lee M. D., Park J. H. (2010). Liver transplantation versus liver resection for the treatment of hepatocellular carcinoma. *Journal of Surgical Oncology*.

[16] Alsemari A., Alkhodairy F., Aldakan A. (2014). The selective cytotoxic anti-cancer properties and proteomic analysis of Trigonella Foenum-Graecum. *BMC Complementary and Alternative Medicine*.

[17] Liu X., Ory V., Chapman S. (2012). ROCK inhibitor and feeder cells induce the conditional reprogramming of epithelial cells. *The American Journal of Pathology*.

[18] Chapman S., Liu X., Meyers C., Schlegel R., McBride A. A. (2010). Human keratinocytes are efficiently immortalized by a Rho kinase inhibitor. *Journal of Clinical Investigation*.

[19] Wang Z., Dabrosin C., Yin X. (2015). Broad targeting of angiogenesis for cancer prevention and therapy. *Seminars in Cancer Biology*.

[20] Khalil M. I. M., Ibrahim M. M., El-Gaaly G. A., Sultan A. S. (2015). Trigonella foenum (Fenugreek) induced apoptosis in hepatocellular carcinoma cell line, HepG2, mediated by upregulation of p53 and proliferating cell nuclear antigen. *BioMed Research International*.

[21] Lall R. K., Syed D. N., Adhami V. M., Khan M. I., Mukhtar H. (2015). Dietary polyphenols in prevention and treatment of prostate cancer. *International Journal of Molecular Sciences*.

[22] Zhang S.-F., Wang X.-L., Yang X.-Q., Chen N. (2014). Autophagy-associated targeting pathways of natural products during cancer treatment. *Asian Pacific Journal of Cancer Prevention*.

[23] Aggarwal B. B., Shishodia S. (2006). Molecular targets of dietary agents for prevention and therapy of cancer. *Biochemical Pharmacology*.

[24] Aboul-Enein B. (2013). Cultural implications of moghat (*Glossostemon bruguieri*) and breastfeeding in Egypt: a brief narrative review. *Online Journal of Cultural Competence in Nursing and Healthcare*.

[25] Amin E. S., Awad O. M. (1968). The mucilage of the roots of clossostemon bruguieri (moghat). *Carbohydrate Research*.

[26] Jacobson M. D., Weil M., Raff M. C. (1997). Programmed cell death in animal development. *Cell*.

[27] Liu X., Zou H., Slaughter C., Wang X. (1997). DFF, a heterodimeric protein that functions downstream of caspase-3 to trigger DNA fragmentation during apoptosis. *Cell*.

[28] Corbiere C., Liagre B., Terro F., Beneytout J.-L. (2004). Induction of antiproliferative effect by diosgenin through activation of p53, release of apoptosis-inducing factor (AIF) and modulation of caspase-3 activity in different human cancer cells. *Cell Research*.

[29] Alhazmi M. I., Hasan T. N., Shafi G., Al-Assaf A. H., Alfawaz M. A., Alshatwi A. A. (2014). Roles of p53 and caspases in induction of apoptosis in MCF-7 breast cancer cells treated with a methanolic extract of nigella sativa seeds. *Asian Pacific Journal of Cancer Prevention*.

[30] Al-Sheddi E. S., Farshori N. N., Al-Oqail M. M., Musarrat J., Al-Khedhairy A. A., Siddiqui M. A. (2014). Cytotoxicity of Nigella Sativa seed oil and extract against human lung cancer cell line. *Asian Pacific Journal of Cancer Prevention*.

[31] Das S., Dey K. K., Dey G. (2012). Antineoplastic and apoptotic potential of traditional medicines thymoquinone and diosgenin in squamous cell carcinoma. *PLoS ONE*.

[32] El-Mahdy M. A., Zhu Q., Wang Q.-E., Wani G., Wani A. A. (2005). Thymoquinone induces apoptosis through activation of caspase-8 and mitochondrial events in p53-null myeloblastic leukemia HL-60 cells. *International Journal of Cancer*.

[33] El-Deiry W. S., Tokino T., Velculescu V. E. (1993). WAF1, a potential mediator of p53 tumor suppression. *Cell*.

[34] Kuo P.-L., Lin C.-C. (2003). Green tea constituent (-)-epigallocatechin-3-gallate inhibits Hep G2 cell proliferation and induces apoptosis through p53-dependent and fas-mediated pathways. *Journal of Biomedical Science*.

[35] Gupta S., Ahmad N., Nieminen A.-L., Mukhtar H. (2000). Growth inhibition, cell-cycle dysregulation, and induction of apoptosis by green tea constituent (-)-epigallocatechin-3-gallate in androgen-sensitive and androgen-insensitive human prostate carcinoma cells. *Toxicology and Applied Pharmacology*.

[36] Choudhuri T., Pal S., Agwarwal M. L., Das T., Sa G. (2002). Curcumin induces apoptosis in human breast cancer cells through p53-dependent Bax induction. *FEBS Letters*.

[37] Liontas A., Yeger H. (2004). Curcumin and resveratrol induce apoptosis and nuclear translocation and activation of p53 in human neuroblastoma. *Anticancer Research*.

[38] Shih A., Davis F. B., Lin H.-Y., Davis P. J. (2002). Resveratrol induces apoptosis in thyroid cancer cell lines via a MAPK- and p53-dependent mechanism. *The Journal of Clinical Endocrinology & Metabolism*.

[39] Raju J., Patlolla J. M. R., Swamy M. V., Rao C. V. (2004). Diosgenin, a steroid saponin of Trigonella foenum graecum (Fenugreek), inhibits azoxymethane-induced aberrant crypt foci formation in F344 rats and induces apoptosis in HT-29 human colon cancer cells. *Cancer Epidemiology Biomarkers and Prevention*.

[40] Mohammad R. Y., Somayyeh G., Gholamreza H., Majid M., Yousef R. (2013). Diosgenin inhibits hTERT gene expression in the A549 lung cancer cell line. *Asian Pacific Journal of Cancer Prevention*.

[41] Han S.-S., Chung S.-T., Robertson D. A., Ranjan D., Bondada S. (1999). Curcumin causes the growth arrest and apoptosis of B cell lymphoma by downregulation of egr-1, C-myc, Bcl-X_L_, NF-*κ*B, and p53. *Clinical Immunology*.

[42] Boehme K., Dietz Y., Hewitt P., Mueller S. O. (2010). Activation of P53 in HepG2 cells as surrogate to detect mutagens and promutagens in vitro. *Toxicology Letters*.

[43] Takawa M., Cho H.-S., Hayami S. (2012). Histone lysine methyltransferase SETD8 promotes carcinogenesis by deregulating PCNA expression. *Cancer Research*.

[44] Bravo R., Macdonald-Bravo H. (1987). Existence of two populations of cyclin/proliferating cell nuclear antigen during the cell cycle: association with DNA replication sites. *The Journal of Cell Biology*.

[45] Fujiwara Y., Komiya T., Kawabata H. (1994). Isolation of a DEAD-family protein gene that encodes a murine homolog of Drosophila vasa and its specific expression in germ cell lineage. *Proceedings of the National Academy of Sciences of the United States of America*.

[46] Scott M., Bonnefin P., Vieyra D. (2001). UV-induced binding of ING1 to PCNA regulates the induction of apoptosis. *Journal of Cell Science*.

[47] Vairapandi M., Azam N., Balliet A. G., Hoffman B., Liebermann D. A. (2000). Characterization of MyD118, Gadd45, and proliferating cell nuclear antigen (PCNA) interacting domains. PCNA impedes MyD118 and Gadd45-mediated negative growth control. *Journal of Biological Chemistry*.

[48] Fujise K., Zhang D., Liu J.-L., Yeh E. T. H. (2000). Regulation of apoptosis and cell cycle progression by MCL1. Differential role of proliferating cell nuclear antigen. *The Journal of Biological Chemistry*.

[49] Hidaka M., Takagi Y., Takano T. Y., Sekiguchi M. (2005). PCNA-MutS*α*-mediated binding of MutL*α* to replicative DNA with mismatched bases to induce apoptosis in human cells. *Nucleic Acids Research*.

[50] Maxwell S. A., Acosta S. A., Tombusch K., Davis G. E. (1997). Expression of Bax, Bcl-2, Waf-1, and PCNA gene products in an immortalized human endothelial cell line undergoing p53-mediated apoptosis. *Apoptosis*.

[51] Xu B., Hua J., Zhang Y. (2011). Proliferating cell nuclear antigen (PCNA) regulates primordial follicle assembly by promoting apoptosis of oocytes in fetal and neonatal mouse ovaries. *PLoS ONE*.

[52] Chang H.-W., Lai Y.-C., Cheng C.-Y., Ho J.-L., Ding S.-T., Liu Y.-C. (1999). UV inducibility of rat proliferating cell nuclear antigen gene promoter. *Journal of Cellular Biochemistry*.

[53] Yu P., Huang B., Shen M. (2001). *p15*
^PAF^, a novel PCNA associated factor with increased expression in tumor tissues. *Oncogene*.

[54] Gulbis J. M., Kelman Z., Hurwitz J., O'Donnell M., Kuriyan J. (1996). Structure of the C-terminal region of p21^WAF1/CIP1^ complexed with human PCNA. *Cell*.

[55] Waga S., Hannon G. J., Beach D., Stillman B. (1994). The p21 inhibitor of cyclin-dependent kinases controls DNA replication by interaction with PCNA. *Nature*.

[56] Savio M., Stivala L. A., Scovassi A. I., Bianchi L., Prosperi E. (1996). P21(waf1/cip1) protein associates with the detergent-insoluble form of PCNA concomitantly with disassembly of PCNA at nucleotide excision repair sites. *Oncogene*.

[57] Bambara R. A., Murante R. S., Henricksen L. A. (1997). Enzymes and reactions at the eukaryotic DNA replication fork. *Journal of Biological Chemistry*.

[58] Agarwal M. L., Taylor W. R., Chernov M. V., Chernova O. B., Stark G. R. (1998). The p53 network. *The Journal of Biological Chemistry*.

[59] Jung H. J., Kim H. L., Kim Y. J., Weon J.-I., Seo Y. R. (2013). A novel chemopreventive mechanism of selenomethionine: enhancement of APE1 enzyme activity via a Gadd45a, PCNA and APE1 protein complex that regulates p53-mediated base excision repair. *Oncology Reports*.

[60] Smith M. L., Ford J. M., Hollander M. C. (2000). p53-mediated DNA repair responses to UV radiation: studies of mouse cells lacking p53, p21, and/or gadd45 genes. *Molecular and Cellular Biology*.

[61] Somasundaram K., El-Deiry W. S. (2000). Tumor suppressor p53: regulation and function. *Frontiers in Bioscience*.

[62] Watanabe H., Pan Z.-Q., Schreiber-Agus N., DePinho R. A., Hurwitz J., Xiong Y. (1998). Suppression of cell transformation by the cyclin-dependent kinase inhibitor p57^KIP2^ requires binding to proliferating cell nuclear antigen. *Proceedings of the National Academy of Sciences of the United States of America*.

[63] Haga N., Fujita N., Tsuruo T. (2005). Involvement of mitochondrial aggregation in arsenic trioxide (As_2_O_3_)-induced apoptosis in human glioblastoma cells. *Cancer Science*.

[64] Scovassi A. I., Poirier G. G. (1999). Poly(ADP-ribosylation) and apoptosis. *Molecular and Cellular Biochemistry*.

[65] Goto M. (2008). Elevation of soluble Fas (APO-1, CD95) ligand in natural aging and Werner syndrome. *Aging*.

[66] Wajant H. (2002). The Fas signaling pathway: more than a paradigm. *Science*.

[67] Qi F., Inagaki Y., Gao B. (2011). Bufalin and cinobufagin induce apoptosis of human hepatocellular carcinoma cells via Fas- and mitochondria-mediated pathways. *Cancer Science*.

[68] Qi F., Li A., Inagaki Y. (2012). Induction of apoptosis by cinobufacini preparation through mitochondria- and Fas-mediated caspase-dependent pathways in human hepatocellular carcinoma cells. *Food and Chemical Toxicology*.

[69] Shao L.-W., Huang L.-H., Yan S., Jin J.-D., Ren S.-Y. (2016). Cordycepin induces apoptosis in human liver cancer HepG2 cells through extrinsic and intrinsic signaling pathways. *Oncology Letters*.

[70] Venturi A., Piaz F. D., Giovannini C., Gramantieri L., Chieco P., Bolondi L. (2008). Human hepatocellular carcinoma expresses specific PCNA isoforms: an in vivo and in vitro evaluation. *Laboratory Investigation*.

[71] Prosperi E. (2006). The fellowship of the rings: distinct pools of proliferating cell nuclear antigen trimer at work. *The FASEB Journal*.

[72] Paunesku T., Mittal S., Protić M. (2001). Proliferating cell nuclear antigen (PCNA): ringmaster of the genome. *International Journal of Radiation Biology*.

[73] Kiya T., Endo T., Goto T. (1998). Apoptosis and PCNA expression induced by prolactin in structural involution of the rat corpus luteum. *Journal of Endocrinological Investigation*.

[74] Liu X., Ye F., Wu J., How B., Li W., Zhang D. Y. (2015). Signaling proteins and pathways affected by flavonoids in leukemia cells. *Nutrition and Cancer*.

[75] Stepanić V., Gašparović A. Č., Trošelj K. G., Amić D., Žarković N. (2015). Selected attributes of polyphenols in targeting oxidative stress in cancer. *Current Topics in Medicinal Chemistry*.

[76] Patil R. H., Babu R. L., Naveen Kumar M. (2015). Apigenin inhibits PMA-induced expression of pro-inflammatory cytokines and AP-1 factors in A549 cells. *Molecular and Cellular Biochemistry*.

[77] Li K.-C., Ho Y.-L., Hsieh W.-T., Huang S.-S., Chang Y.-S., Huang G.-J. (2015). Apigenin-7-glycoside prevents LPS-induced acute lung injury via downregulation of oxidative enzyme expression and protein activation through inhibition of MAPK phosphorylation. *International Journal of Molecular Sciences*.

[78] Arango D., Diosa-Toro M., Rojas-Hernandez L. S. (2015). Dietary apigenin reduces LPS-induced expression of miR-155 restoring immune balance during inflammation. *Molecular Nutrition and Food Research*.

[79] Shukla S., Gupta S. (2010). Apigenin: a promising molecule for cancer prevention. *Pharmaceutical Research*.

[80] Ma X., Li Y.-F., Gao Q. (2008). Inhibition of superoxide anion-mediated impairment of endothelium by treatment with luteolin and apigenin in rat mesenteric artery. *Life Sciences*.

[81] Jeong Y.-J., Choi Y.-J., Choi J.-S. (2007). Attenuation of monocyte adhesion and oxidised LDL uptake in luteolin-treated human endothelial cells exposed to oxidised LDL. *British Journal of Nutrition*.

[82] Popa O., Băbeanu N. E., Popa I., Niță S., Dinu-Pârvu C. E. (2015). Methods for obtaining and determination of squalene from natural sources. *BioMed Research International*.

[83] Kedl J. D., Kedl R. M. (2015). How squalene GLAdly helps generate antigen-specific T cells via antigen-carrying neutrophils and IL-18. *European Journal of Immunology*.

[84] Chen Z., Kong S., Song F., Li L., Jiang H. (2012). Pharmacokinetic study of luteolin, apigenin, chrysoeriol and diosmetin after oral administration of Flos Chrysanthemi extract in rats. *Fitoterapia*.

[85] Dewé W., Durand C., Marion S., Oostvogels L., Devaster J., Fourneau M. (2015). A multi-criteria decision making approach to identify a vaccine formulation. *Journal of Biopharmaceutical Statistics*.

[86] Tegenge M. A., Mitkus R. J. (2015). A first-generation physiologically based pharmacokinetic (PBPK) model of alpha-tocopherol in human influenza vaccine adjuvant. *Regulatory Toxicology and Pharmacology*.

[87] Ezhilan B. P., Neelamegam R. (2012). GC-MS analysis of phytocomponents in the ethanol extract of *Polygonum chinense* L.. *Pharmacognosy Research*.

[88] Kelly G. S. (1999). Squalene and its potential clinical uses. *Alternative Medicine Review*.

[89] Pandino G., Lombardo S., Moglia A., Portis E., Lanteri S., Mauromicale G. (2015). Leaf polyphenol profile and SSR-based fingerprinting of new segregant *Cynara cardunculus* genotypes. *Frontiers in Plant Science*.

[90] Alshatwi A. A., Shafi G., Hasan T. N., Syed N. A., Khoja K. K. (2013). Fenugreek induced apoptosis in breast cancer MCF-7 cells mediated independently by fas receptor change. *Asian Pacific Journal of Cancer Prevention*.

[91] Shabbeer S., Sobolewski M., Anchoori R. K. (2009). Fenugreek: a naturally occurring edible spice as an anticancer agent. *Cancer Biology and Therapy*.

